# Comparing covariation among vaccine hesitancy and broader beliefs within Twitter and survey data

**DOI:** 10.1371/journal.pone.0239826

**Published:** 2020-10-08

**Authors:** Sarah A. Nowak, Christine Chen, Andrew M. Parker, Courtney A. Gidengil, Luke J. Matthews

**Affiliations:** 1 Department of Pathology and Laboratory Medicine, Larner College of Medicine at the University of Vermont, Burlington, Vermont, United States of America; 2 RAND Corporation, Boston, Massachusetts, United States of America; 3 Pardee RAND Graduate School, Santa Monica, California, United States of America; 4 RAND Corporation, Pittsburgh, Pennsylvania, United States of America; 5 Boston Children’s Hospital, Boston, Massachusetts, United States of America; University of Pittsburgh, UNITED STATES

## Abstract

Over the past decade, the percentage of adults in the United States who use some form of social media has roughly doubled, increasing from 36 percent in early 2009 to 72 percent in 2019. There has been a corresponding increase in research aimed at understanding opinions and beliefs that are expressed online. However, the generalizability of findings from social media research is a subject of ongoing debate. Social media platforms are conduits of both information and misinformation about vaccines and vaccine hesitancy. Our research objective was to examine whether we can draw similar conclusions from Twitter and national survey data about the relationship between vaccine hesitancy and a broader set of beliefs. In 2018 we conducted a nationally representative survey of parents in the United States informed by a literature review to ask their views on a range of topics, including vaccine side effects, conspiracy theories, and understanding of science. We developed a set of keyword-based queries corresponding to each of the belief items from the survey and pulled matching tweets from 2017. We performed the data pull of the most recent full year of data in 2018. Our primary measures of belief covariation were the loadings and scores of the first principal components obtained using principal component analysis (PCA) from the two sources. We found that, after using manually coded weblinks in tweets to infer stance, there was good qualitative agreement between the first principal component loadings and scores using survey and Twitter data. This held true after we took the additional processing step of resampling the Twitter data based on the number of topics that an individual tweeted about, as a means of correcting for differential representation for elicited (survey) vs. volunteered (Twitter) beliefs. Overall, the results show that analyses using Twitter data may be generalizable in certain contexts, such as assessing belief covariation.

## Introduction

Over the past decade, the percent of adults in the United States who use some form of social media has roughly doubled, increasing from 36 percent in early 2009 to 72 percent in 2019 [1]. There has been a corresponding increase in research aimed at understanding opinions and beliefs that are expressed online [2]. Social media also provides a way for researchers to observe the beliefs of a greater number of individuals than is possible through more traditional research methods, such as surveys and focus groups. On the other hand, users of any given social media platform are not representative of the population as a whole, views are volunteered rather than elicited, and the generalizability of findings from social media research is a subject of ongoing debate [3, 4].

Social media and other online platforms are frequently cited as sources of misinformation about vaccines and vaccine hesitant beliefs [5–7]. Despite overwhelming evidence about the safety and efficacy of childhood vaccines [8], vaccination rates often fall below targets [9]. Scientific understanding of how vaccines work does not appear to be necessary for parents to fully vaccinate their children [10]. Attempts to increase parents’ scientific understanding of how vaccines work to increase their willingness to fully vaccinate their children can be ineffective and even *increase* vaccine hesitancy [11]. This suggests that vaccine hesitancy may result from broader social and cognitive processes beyond understanding and acceptance of scientific evidence around the safety and efficacy of vaccines.

Our primary research question was whether Twitter and survey data measuring vaccine hesitancy and a broader set of beliefs covary in similar ways. We note that the conclusions drawn from an analysis of any data set depends on both underlying data and on how the data are processed and analyzed. This is particularly true of unstructured text such as social media data. For that reason, we examined whether different approaches to processing the Twitter data influenced the agreement between the Twitter and survey analyses. These processing steps were: removing bots, inferring the stance, and resampling the Twitter data based on the number of topics an individual tweeted about. The motivation behind resampling was that one key difference between Twitter and survey data is that tweets on Twitter are the result of an individual taking the initiative to post, respond, retweet, or share an article on a particular topic. In other words, beliefs expressed on Twitter are volunteered. In contrast, survey beliefs are elicited using the same set of questions for all respondents. As a result, we might expect the distribution of the number of topics tweeted about by individuals in our data set to be very different from the distribution of topics individuals express strong beliefs about on a survey. We found that single-topic accounts or accounts that tweet about only a limited number of topics are over-represented in Twitter data relative to the numbers of individuals who express strong beliefs about a limited number of topics in the general population based on our survey results. Therefore, we hypothesized that resampling the Twitter data so that the distribution of the number of topics tweeted about matched the distribution of the number of strong beliefs in our survey data would make Twitter data more representative of the general population, which would improve the agreement between the survey and Twitter data analyses.

We found very good qualitative and quantitative agreement between the first principal component of the principal component analysis (PCA) using survey and Twitter data after removing bots and inferring, based on manually coded weblink domains, whether individuals agreed or disagreed with the topic they tweeted about (stance). Resampling the Twitter data so that the distribution of the number of beliefs matched that from the survey further improved agreement.

## Materials and methods

This study was approved by the RAND Institutional Review Board, called the RAND Human Subjects Protection Committee (HSPC). The approval letter stated: "On 9/12/2017, the Human Subjects Protection Committee (HSPC) approved the study referenced below in expedited review Category 7. In addition, the HSPC approved a waiver of documentation of consent under 45 CFR 46.117(c) [2]. The HSPC determined that the study is minimal risk." While documentation of consent was not required, participants in the RAND American Life Panel consent to participate in the panel through an annual consent process. Those who do not consent are not included in the survey panel.

### Survey

We conducted a survey through the RAND American Life Panel, which is an online panel of adults ages 18 and older living in the United States. The ALP uses a probability-based sampling method of the US population that includes individuals regardless of whether they are online or not. ALP provides panelists who are not already online by providing home internet access to individuals without regular internet access who agree to participate in the panel. We invited 716 members of the panel with children under the age of 21 years to take the survey, and we received 615 responses (overall completion rate 85%). We asked about knowledge and beliefs on 35 items, which included: 8 political conspiracies, health conspiracies, 9 putative vaccine side effects, 2 gestalt vaccine endorsements, and 6 items about vaccine biology/epidemiology.

All belief survey responses were on a Likert scale from 0 to 6 where “0” indicated “Strongly disagree is true”, 3 indicated “Undecided or not sure”, and 6 indicated “Strongly agree is true. We include the full list of survey items in S1 Table.

### Twitter data

We pulled tweets that were posted in 2017 about topics corresponding with the 35 belief items from our prior survey, because this was the most recent complete year as of summer 2018, when we pulled the data. We conducted our data pulls using Gnip, a major vendor of Twitter data that enables historical data pulls with keyword and other criteria for inclusion. The first step in acquiring the Twitter data for our analysis was to develop a set of 35 Twitter search queries (hereafter “tags”) corresponding to the 35 belief items from our prior survey. After developing an initial set of 35 queries, we pulled a preview of tweets posted on a single day that matched our queries. There was wide variation in the number of tweets we received on each query term; some queries resulted in no matching tweets while a “deep state” query resulted in over 18,000 tweets in a one-day preview. We used an iterative refinement process in which we reviewed up to 20 tweets returned in the preview for each tag. If we observed false positives in the samples we reviewed, we refined our query terms for that tag to be more specific. False positives were tweets clearly about a different topic than the belief we intended, for example, tweets about household cleaning products when we were searching for chemtrail conspiracy beliefs. For tags with no or very low single-day volume (<10/day), we broadened our query terms. We additionally included relevant hashtags identified in our reviews of tweets. For example, we included #vaccineswork as a term in our queries about vaccine endorsement. At this phase, our queries were intended to identify only tweets about a topic, but not identify the tweet’s stance (agreement or disagreement with the belief). For example, tweets about the false belief that Barack Obama was not born in the United States are at times sent both by those endorsing this belief and by those critiquing it, but all such tweets would be about “birtherism” and would be picked up by the birtherism search query tag.

There were 5 belief items for which we could not identify a set of query terms that were specific enough to give us a low false-positive rate, but general enough to result in any hits. These were “CIA Created HIV”, “Vaccine Spread HIV Africa”, “CIA Bin Laden Vaccination”, “CDC Hides Side Effects”, and “Vaccine Profit Motive”. The full list of belief items from the survey are shown in S1 Table. In addition, we did not have a query for the belief that the HPV vaccine can cause sterility (“HPV Vacc Causes Sterility”); but, we had a query for the more general belief that vaccines can cause sterility (“Vaccines Created to Sterilize”). These were separate beliefs in our survey, but were covered by the same terms in our Twitter query. As a result, we had 29, rather than 35, belief items in our Twitter query. The final query terms used to pull the data are listed in S1 Table. Tweets that matched a specific query were given a “belief tag” describing the corresponding belief. In some cases, tweets were tagged with multiple beliefs.

The Twitter query returned a total of 8,829,987 tweets generated by 1,350,451 unique users. We removed accounts that tweeted once during the sampling time frame as a low threshold to restrict our analysis to engaged accounts. This resulted in 8,064,173 tweets from 584,636 users in the sample.

Next, we used the Bot-o-meter API [12, 13] to identify accounts that were likely to be bots. The Bot-o-meter is a machine learning algorithm trained to predict the likelihood that a user is a bot based on the user’s profile, friends, social network structure, temporal activity patterns, language, and sentiment. The models also calculate a Complete Automated Probability (CAP)—the probability of an account being completely automated, by taking into account the estimated overall prevalence of bots [12, 13]. We used a CAP threshold of 0.5 to remove tweets from our data set that the tool determined to have at least a 50 percent likelihood of being a bot. The final de-botted file had 7,578,547 tweets from 551,738 unique accounts. We note that because we pulled historical data from Gnip, our data did not contain tweets from suspended accounts [14], including Russian bots and trolls that were found to amplify the vaccine debate [15]. Recognizing that bot detection is imperfect, we conducted a sensitivity analysis in which we used the stricter criteria of only including accounts with a less than 20 percent likelihood of being a bot (CAP<0.2).

### File pairs

We created four pairs of data files; one member of each data file pair was derived from our survey data, and one was derived from the Twitter data. These file pairs enabled us to examine how different data processing steps affected whether similar conclusions about belief covariation could be drawn from Twitter and survey data. We first will give an overview of each data file pair including its analytic purpose and file contents. Following this overview, we present a more detailed description of how each file in the pair was created from the original survey and Twitter data files. S1 Fig in the supplementary material presents a flow diagram depicting the relationships among the different analytic files that we created.

### File pair overview

1. **Topic data file pair.** The purpose of this data file pair was to examine the agreement between Twitter and the survey data with only minimal processing of the Twitter data. For this pair, the Twitter data were processed to be a 1 if the account tweeted about a topic and otherwise 0. To make a conceptually comparable transform of the survey data, for this pair, the survey data were processed to be a 1 if respondents selected the most extreme boxes on either the *agree* or *disagree* end of the scale. All other selections were set to 0. We also conducted a sensitivity analysis in which any non-neutral survey response was coded to be 1 and only the neutral response (3 on the 0 to 6 Likert scale) was coded as 0.
2. **Stance data file pair.** The purpose of this data file pair was to examine whether inferring tweet stance (i.e. agreement or disagreement with the topic tweeted about) improved the concordance between Twitter and survey data. We inferred stance on 10 belief tags by manually coding websites commonly linked to by the five most commonly tweeted about vaccine beliefs and the five most commonly tweeted about political or conspiracy beliefs. Data elements in these files could take on values 0 (neutral stance), -1 (negative), or +1 (positive). For the Stance data file pair, the survey data were processed such that individuals who selected the most extreme disagree box were -1, the most extreme agree box were 1, and all others were 0. We also conducted a sensitivity analysis in which any agree response to the survey was coded as +1 and any disagree response was coded as -1.
3. **Limited topic file pair.** The purpose of this data file pair was to serve as a robustness check on any conclusions that we could draw from a comparison of the analysis of the Topic and Stance data file pairs. The Topic and Stance data file pairs differ in three ways. First, the Stance data file has information about whether respondents/tweeters agreed or disagreed with the topic. The Topic file pair does not have agree/disagree information. Second, the Stance data file pair contains fewer belief items than the Topic data file pair because we inferred stance for only 10 of 29 belief items. Third, the Stance data file pair contains fewer individuals since we dropped survey respondents and tweeters for whom the inferred stance on all 10 belief items was zero. The limited Topic file pair contains only the belief items and observations that are included in the stance file so that the only difference between the Limited Topic and Stance file pairs is the inference of stance.
4. **Resampled stance file pair.** We created a file pair in which Twitter data with stance inferred, was re-sampled so that the distribution of the number of topics about which individuals expressed strong beliefs was similar to that found in the survey data. The survey data for this file pair are identical to those described for the Stance file pair.

Table 1 shows an overview of the four data file sets.

**Table 1:** Description of data file Pairs.

Data file pair	Purpose of data file pair	Values Data Could Take	Number of belief tags	Number of Rows (Individuals) in Survey File	Number of Rows (Accounts) in Twitter File
Topic	Examine agreement with minimal processing of Twitter data	0, +1	29	565	551,738
Stance	Examine the impact of inferring stance with Twitter data	-1, 0, +1	10	550	183,169
Limited Topic	Create a file without stance that is otherwise identical to the Stance data files to isolate the effect of inferring stance	0, +1	10	550	183,169
Resampled Stance	Examine whether the number of belief items tweeted about could be used to re-sample Twitter data to make the data more representative of the population	-1, 0, +1	10	550	550

#### Topic survey file

To create the Topic survey data file, we coded any response of “Strongly Disagree is True” or “Strongly Agree is True” (0 or 6) as a “1,” indicating that the survey respondent had a strong belief about the item and coded the response as “0” otherwise. We coded the data in this way to attempt to create a file that would be comparable to the Twitter file indicating if an account tweeted about a particular topic, using the assumption that individuals are most likely to tweet about topics about which they have a strong opinion. For example, political tweets represent points of view that are more extreme than those held by the public in general [16]. We test the impact of this assumption in a sensitivity analysis in which any agree or disagree survey response (i.e., any non-neutral response) is coded as “1” in the topic survey file.

#### Topic Twitter file

We took the tweet-level debotted file described above and created a file in which each row represented a single Twitter user in our data, and columns were belief tags. Data elements in the file could take on values of “0” if an account did not have any tweets tagged with the belief in our data and “1” if the account did.

#### Survey stance file

For the survey stance file, a response of “Strongly Disagree is True” was coded as “-1”, “Strongly Agree is True” was coded as +1, and other responses were coded as “0”. We tested the impact of this assumption in a sensitivity analysis in which any agree survey response was coded as “+1” and any disagree survey response became “-1” in the survey stance file.

#### Twitter stance file

We developed a procedure to infer the stance of tweets using the website domain information included in the tweets. Given that our data set had over 500,000 tweets, manually coding the stances of each tweet was not feasible. However, we hypothesized that we could reliably infer a user’s stance on particular beliefs based on assessing the stances of websites he or she linked to in tweets. To use this link information as efficiently as possible, we assessed website stance at the host level (for example, www.cdc.gov) rather than at the individual page level (for example, https://www.cdc.gov/vaccines/parents/tools/parents-guide/parents-guide-part4.html). We tested this hypothesis using the 5 most common political or conspiracy-related belief tags and the 5 most common vaccine-related belief tags. These were: “Deep State”, “9/11 Inside Job”, “JFK Assassination”, “Chemtrails”, and “Birtherism”, “Vaccines Cause SIDS”, “Drs Hide Side Effects”, “Vaccines Benefit Public”, “Vaccines Cause Asthma”, and “MMR Autism.

We used the parseURI function from the XML package in R [17] to parse the full urls referred to in the tweet links and extract the host name. For example, we extracted the host www.cdc.gov from the full url: https://www.cdc.gov/vaccines/parents/tools/parents-guide/parents-guide-part4.html. We identified the top 25 host names that were most commonly linked in tweets tagged with “Deep State,” the top 25 hosts linked to in the other 4 political/conspiracy tags, and the top 25 hosts appearing in tweets tagged with health/vaccine-related beliefs. We examined the top 25 hosts from deep state tweets separately from other urls because the “Deep State” tag was far more common than other tags. As a result, the top 25 hosts from all political/conspiracy tags were entirely determined by the top 25 hosts from “Deep State” tags. Two authors of this paper (SAN and LJM) independently coded the stances of these host websites on each of the corresponding topic beliefs as “agree” (+1) “neutral” (0) or “disagree” (-1). We then used our consensus rating of the host stance to infer the stance of any tweet containing a link to that host and tagged with a particular belief.

We validated the website-based inference of tweet stance by manually coding the stances of 20 randomly selected tweets for each of the 10 belief tags included in this part of our analysis.

#### Limited topic survey file

For the Limited Topic survey data file, as with the Topic survey data file, we coded any response of “Strongly Disagree is True” or “Strongly Agree is True” (0 or 6) as a “1” indicating that the survey respondent had a strong belief about the item and coded the response as “0” otherwise. We then included only the belief items for which we inferred stance in the Twitter data.

#### Limited topic Twitter file

We created the Limited Topic Twitter file by taking the absolute value of the Twitter Stance file. In other words, data elements that were either +1 or -1 from the Twitter Stance file became +1 in the Limited Topic Twitter file.

#### Resampled stance survey file

The survey file used for the Resampled Stance comparative analysis was the same as the Stance survey file.

#### Resampled stance Twitter file

We re-sampled the Twitter Stance data file so that the distribution of the number of topics individuals tweeted about was the same as the distribution of the number of topics an individual expressed a strong belief about (i.e., belief value was +1 or -1) from the Stance survey file. Table 2 shows the distribution of the number of non-zero belief tags in the Twitter and Survey stance files before resampling. Note the large number of single-stance Twitter accounts relative to single-stance survey respondents. Resampling was designed to correct for this. When we re-sampled, each row (account) in the Twitter stance file was categorized as tweeting about 1, 2, 3, 4 or 5+ topics. To create the re-sampled Twitter stance file, we randomly selected 68 rows (the number of single-topic rows from the survey stance file) from the twitter stance file that tweeted about 1 topic to include in the re-sampled file, 67 that tweeted about 2 topics, and so on.

**Table 2:** Distribution of non-zero belief tags in Twitter and survey stance files (before resampling).

Number of topics	Twitter stance file, count	Survey stance file, count	Twitter stance file, percent	Survey stance file, percent
1	166216	68	90.7%	12.4%
2	13432	67	7.3%	12.2%
3	2303	73	1.3%	13.3%
4	717	76	0.4%	13.8%
5+	501	266	0.3%	48.4%

### Sensitivity analyses

We conducted three sensitivity analyses whose results appear in S5 and S6 Tables. The first was an alternate survey coding. In the main results, we code only the “Strongly agree is true” (6 on a Likert scale) and “Strongly disagree is true” (0 on a Likert scale) survey responses as non-neutral. In the alternate survey coding, we code all *agree* statements (4, 5, or 6 on the Likert scale) and all *disagree* statements (0, 1, or 2 on the Likert scale) as non-neutral. The second sensitivity analysis was a strict bot removal in which we kept only accounts with a Complete Automated Probability (CAP) <0.2 instead of CAP <0.5, which corresponds to keeping accounts with less than a 20% probability of being a bot. In the third analysis, we re-sampled the Twitter data 100 times for the resampled stance data file pair.

### Principal Component Analysis (PCA)

We ran principal component analysis (PCA) on our data in R using the prcomp function from the stats package [18]. For all of our analyses, we ran the PCA on centered, scaled data. We tested the similarity between the first principal components in two ways. First, we compared the PC1 loadings. We examined the qualitative agreement in the Twitter and survey PC1 loadings by comparing which items loaded in the same direction (positive or negative) in the two PC1s. We also calculated the correlation between the PC1 loadings estimated using the survey (**D**_S_) and Twitter data (**D**_T_), which is a method that has been used to compare factor loadings [19]. Comparing the loadings gives insight into how comparable two PCAs are in terms of *covariation among the belief items* in the data set. We also used a method described in Jolliffe 2003 [20], which compares PC scores estimated from two different PCAs. This method gives insight into how comparable the two PCAs are in describing *variation among the observations* in the data.

To compare the PC1 scores derived from the Twitter and survey data, we estimated the PCA transformation using both the survey data—we call this projection **P**_S_—and the Twitter data. The Twitter projection is **P**_T_. These PCA transformations are characterized by the loadings of each of the original variables on the first principal component from either the survey or Twitter data. We then projected both the Twitter data and the survey data into the new PCA coordinate basis using both P_S_ and P_T_. In other words, we used the loadings from PC1 as derived from the survey data to calculate predicted PC1 scores for the Twitter data, and likewise we calculated PC1 scores for survey data from the PC1 loadings estimated from the Twitter data. Mathematically, we calculated (**P**_S_**D**_S_)_*,1_; (**P**_T_**D**_S_)_*,1_; (**P**_T_**D**_T_)_*,1_; and (**P**_S_**D**_T_)_*,1_. The subscript “*,1” indicates the first column of a resulting matrix. We then used linear regression to estimate the models

$${\left(P_{S}D_{S}\right)}_{*,1}=\beta \;{\left(P_{T}D_{S}\right)}_{*,1}+c+\varepsilon_{i}$$

and

$${\left(P_{T}D_{T}\right)}_{*,1}=\beta \;{\left(P_{S}D_{T}\right)}_{*,1}+c+\varepsilon_{I,}$$

where β is a coefficient estimated in the model and c is a constant estimated in the model. The R squared in the estimated models provides a measure for the similarities between the first principal components of the projections **P**_S_ and **P**_T_ [20]. It is the proportion of variance from one set of PC1 scores that explains the variance in the other.

## Results

### Inter-rater reliability and stance validation

We inferred tweet stance by manually coding the stance of common website domains linked to in tweets. The stance of a tweet containing a weblink to a coded domain was assigned the stance of domain. To validate this approach, two authors of this paper, SAN and LJM, independently coded the stances of 20 tweets from each topic included in the stance data files. The average interrater reliability for tweet coding across the ten tags was 86% agreement, Cohen’s kappa was 0.55. Tag-level results are shown in S3 Table. For two tags, all tweets were coded with the same stance and the Cohen’s kappa was therefore undefined. These tags were included in the calculation of the average percent agreement, but excluded from the calculation of the mean Cohen’s kappa. For each coder, we compared the stances assigned directly to the tweets to the stances inferred using weblinks. The average agreement between coded and inferred tweet stance was 67% across all tags, and average Cohen’s kappa was 0.45. Tag-level results are shown in S4 Table. When we inferred stance in the Twitter data, we found that of the 7,578,547 tweets in the de-botted file, 3,539,561 (47%) contained a url, and 432,011 out of 551,738 (78%) of accounts had a least one tweet with a url. When we restricted the data set to tweets tagged with the 10 belief items for which we inferred stance and tweets for which we inferred a non-neutral stance, the resulting file had 909,479 tweets from 183,169 accounts.

### Belief frequencies in the data file pairs

**Fig 1:**
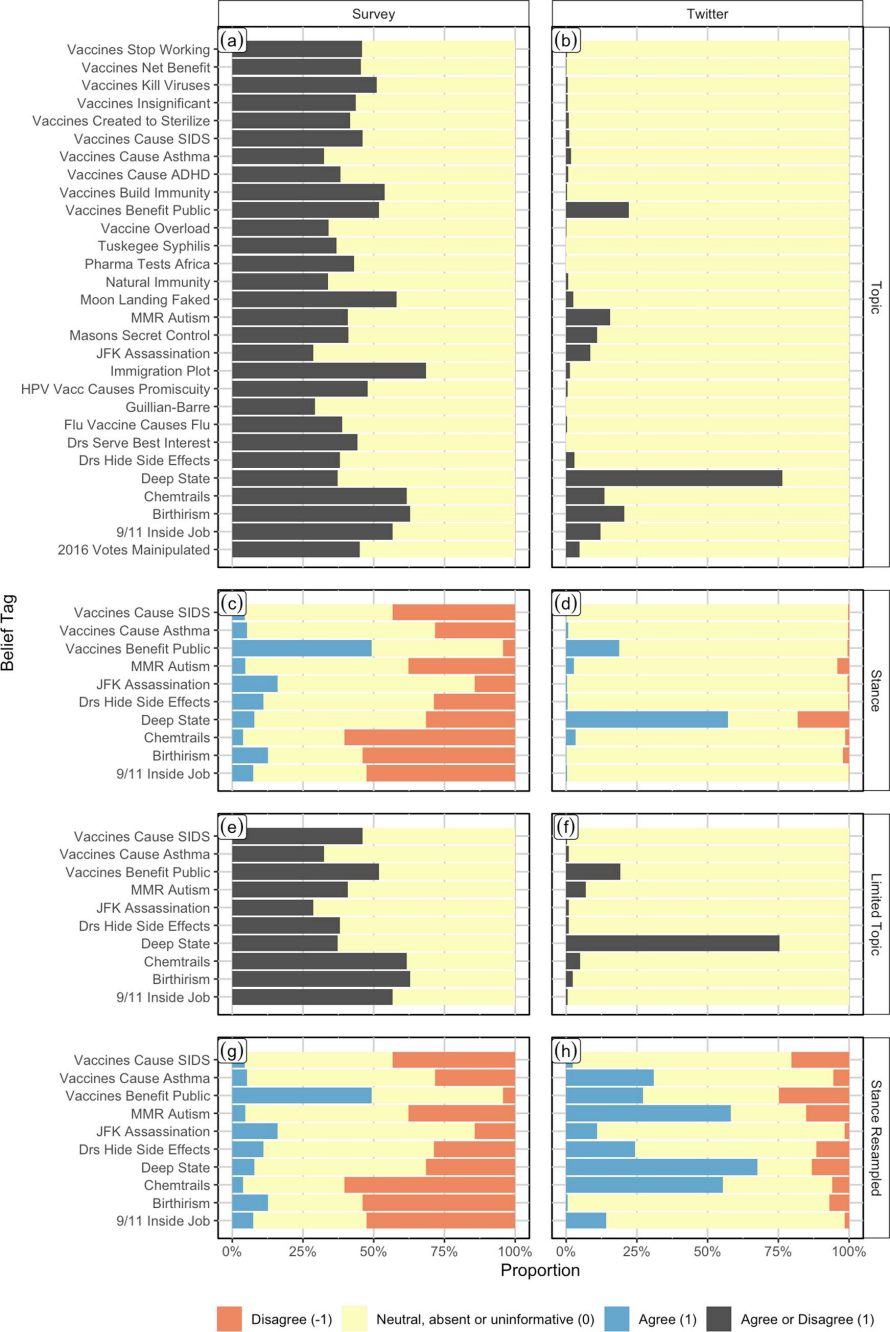
Proportion of individuals or accounts with different belief tag values in the survey and Twitter data files for each of the four data file pairs. In panels a, b, e, and f, black indicates presence (1) and yellow absence (0) of a belief. Within panels c, d, g, and h, blue indicates agreement (+1), yellow absence (0), and orange disagreement (-1) with a belief.

Fig 1 shows the percentages of individuals or accounts with different values in the Twitter and survey files in each of our file pairs. In the survey Topic data file (Fig 1A), the percentage of individuals expressing strong beliefs (black bars) fell between 25% and 75%; there were no items for which a very high or very low percentage of individuals expressed strong beliefs. In contrast, in the Twitter data (Fig 1B), we found that in our sample, which included individuals who tweeted at least twice about any of our belief items, most items were discussed by fewer than 25% of our sample. The “Deep State” belief tag was by far the most common in the Twitter data set and was tweeted about by approximately 75% of individuals. The low frequencies of most belief tags in our data set is largely a reflection of the fact that many accounts that tweet about “Deep State” do not tweet about any other belief tag.

When we inferred stance in the Twitter data (Fig 1D), we found that “Deep State” and “Vaccines Benefit Public” remained the most common beliefs. Over half of individuals in this data set expressed agreement with the “Deep State” belief tag (blue bar), while around 20% expressed disagreement (orange bar). In addition, around 25% of accounts in this data set express agreement with “Vaccines Benefit Public” and a very small percentage expressed disagreement. When we re-sampled the Twitter data so that the distribution of the numbers of items that individuals expressed beliefs about was similar to the distribution we observed in the survey (Fig 1H), we found that in addition to the “Deep State” belief tag, over half the sample expressed agreement with “MMR Autism” (a link between vaccines and autism) and “Chemtrails” (a belief that airplane contrails contain chemical or biological agents that are released into the atmosphere as part of secret government programs).

We now turn to the results of our PCA. We focus on the results for the first principal component because scree plots from the survey PCAs suggested that there was one dominant principal component. While the scree plot for the Twitter topic data file suggested that up to four principal components were important, after stance inference and resampling, the Twitter scree plot suggested a single dominant principal component. Scree plots for all analytic files used in the analysis are shown in S2–S5 Figs.

### Comparison of first principle component loadings

**Fig 2:**
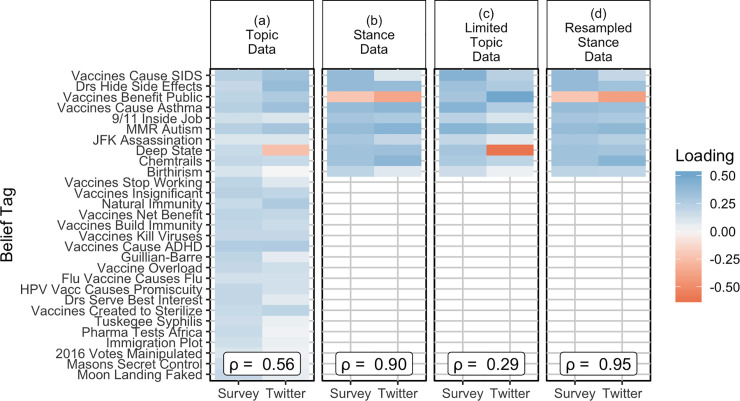
Loadings on PC1, the first principal component from the PCA analysis for each data file. Values of ***ρ*** are the Pearson’s correlation coefficient for each pair of PC1 loadings.

Fig 2 shows the loadings of the first principal component from the PCA. All belief items loaded in the same direction on the first principal component in the Topic survey data (Fig 2A). This suggests that some people tend to use the extreme ends of the scale when responding to the survey, which would result in those individuals having most or all items coded as “1”, while others tend to answer towards the middle of the scale. When we conducted the principal component analysis with the comparable Twitter data set, we found that all items with the exception of the “Deep State” belief tag loaded in the same direction, suggesting that the first principal component captured the tendency of people to either tweet about the “Deep state,” or to tweet about other topics. The first principal component in the Twitter and survey Topic data sets appeared to describe qualitatively different phenomena. When we incorporated stance information (Fig 2B), we found that for both the survey and Twitter data, all items loaded in the same direction with the exception of the “Vaccines Benefit Public” belief. All other beliefs examined were conspiracy theories and unproven or disproven negative beliefs about vaccines. In this case, the results of the principal component analysis using both the survey and Twitter information had similar qualitative interpretations. The results of the Limited Topic data analysis (Fig 2C) were qualitatively similar to the results of the Topic data analysis; and, the results of the Resampled Stance analysis (Fig 2D) were qualitatively similar to the results of the Stance data set analysis.

### Comparison of first principle component scores

**Fig 3:**
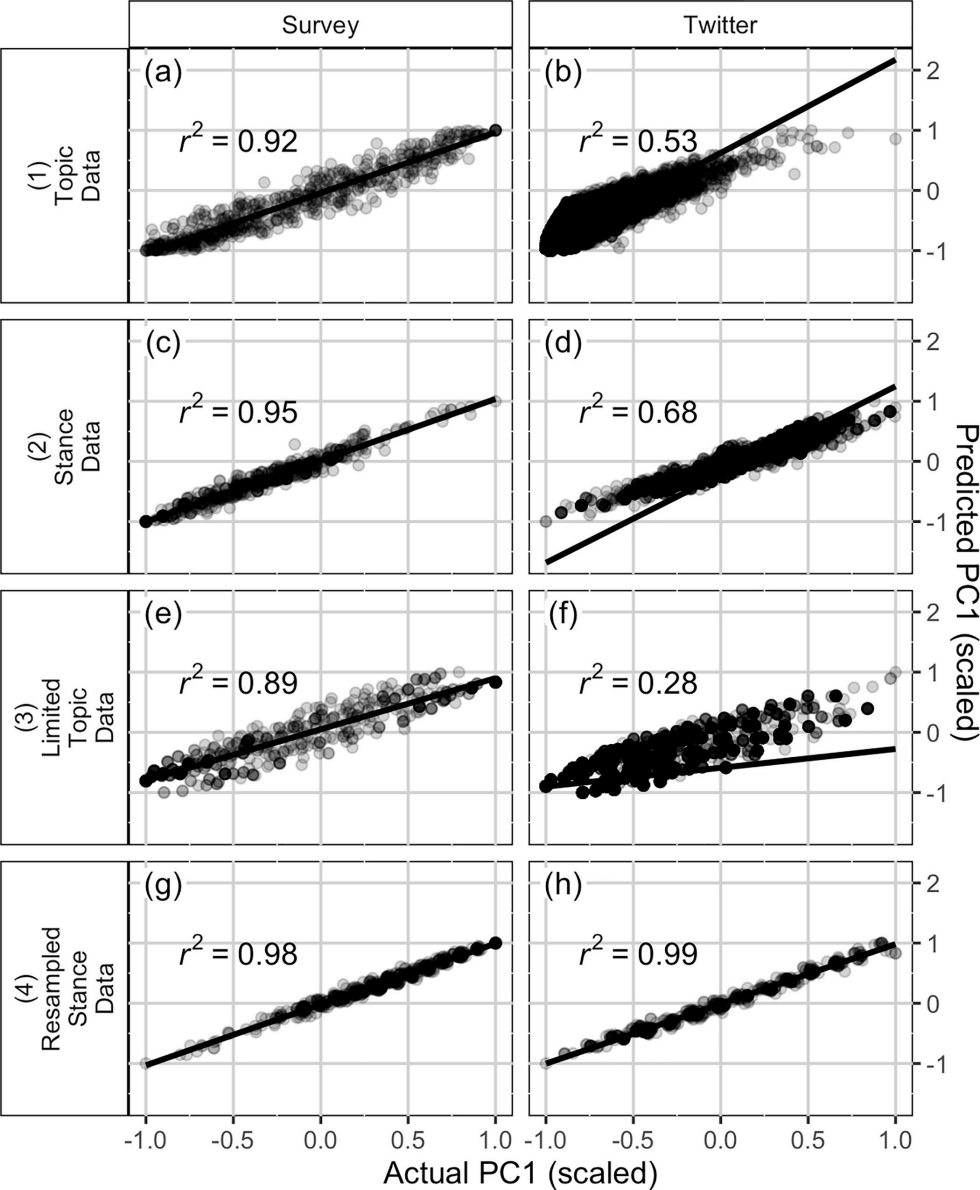
Scatter plot and linear fit of predicted PC1 score and actual PC1 score. The “actual PC1 score” was calculated using the loadings from the data set for which we were estimating the scores. I.e., actual scores for Twitter data were calculated using Twitter PC1 loadings and actual scores for survey data were calculated using survey PC1 loadings. The “predicted PC1 score” for Twitter data sets was calculated using survey PC1 loadings and the “predicted PC1 score” for survey data sets was calculated using Twitter PC1 loadings. For each plot, the predicted and actual PC1 scores were re-scaled to lie between -1 and +1, which did not alter the correlation.

Fig 3 shows the results of the linear regressions that describe the association between the actual PC1 scores for each data set (survey or Twitter) and predicted PC1 scores calculated using PC1 loadings from the other data set (Twitter or survey). We found that the agreement between PC1 scores was highest when stance information was included in the analysis (Fig 3C, 3D, 3G and 3H) compared to when it was not (Fig 3A, 3B, 3E and 3F). In addition, we found that for the Topic, Stance, and Limited Topic data pairs (Fig 3A–3F) predictions for survey PC1 values using the Twitter PCA analysis were much better than predictions of Twitter PC1 values using the survey PCA analysis. However, when we re-sampled the Twitter stance data set so that the distribution of the numbers of items on which individuals expressed strong beliefs was more similar to the distribution from the survey data (Fig 3G and 3H), we found improvement–that Twitter PC1 values were now predicted very well by the survey PCA. This resampling procedure reduced the number of individuals who tweeted about only a small number of topics; recall from Table 2 that 90.7% of accounts in the Twitter stance file tweeted about only a single topic. We resampled the data so that 12.4% of observations in the resampled Twitter stance file tweeted about only one topic. The fact that predictions of PC1 scores for the Twitter data improved markedly after the resampling procedure suggests that it is more difficult to predict PC1 scores for individuals who tweet about a limited number of topics than those who tweet about several. This makes sense given that the PC1 value for an individual who tweets about a single topic is the PC1 loading for just that topic. Overall, we find very good qualitative and quantitative agreement for the first principal component of the PCA using the survey and Twitter data in the data sets that contain stance information and where the Twitter data is re-sampled to better match the “number of topics” distribution we observe in the survey data.

## Discussion

We used principle components analysis to measure belief co-variation in Twitter data and nationally representative survey and assessed the similarity in the loadings and scores obtained using the first principle component (PC1) of both analyses. We compared PC1 using four pairs of data, corresponding to different ways of processing the data, and in particular, different ways of extracting meaning from Twitter data. Despite the fact that belief and stance frequencies were very different in our two data sets, we found very good agreement in our analysis of belief covariation using the Twitter and survey data when we used urls linked in tweets to infer tweet stance. We found that resampling Twitter data according to the number of topics on which users expressed strong beliefs, using the distribution of number of beliefs from the survey as a target, we further improved the belief covariation agreement between the Twitter and survey data sets.

It is not surprising that we find different frequencies of beliefs using Twitter compared to using the survey. It is well known that Twitter users in the US are not representative of the broader US population. A recent Pew study found that Twitter users are younger, more educated, and wealthier than the US population as a whole [21]. In addition, on Twitter we see only what individuals choose to tweet about. Tweets arise from selected individuals within the population, and perhaps more importantly tweets are a selective public performance by these individuals. If individuals do not tweet on a particular topic, for the purposes of our analysis we infer that they have a neutral stance on the topic. However, it is possible that they do have a strong opinion on the topic, but simply chose not to tweet about it. For example, in our survey data we found that over half of survey respondents strongly disagree with the idea of “chemtrails” while fewer than 10 percent strongly agreed. However, we saw many more individuals with an “agree” stance on chemtrails in the resampled Twitter stance data than with a disagree stance (56% agree vs. 6% disagree). This suggests that supporters of the idea of “chemtrails” are much more vocal on Twitter than are individuals who disagree with “chemtrails”. We note that because we only inferred stance on tweets with a link to certain commonly linked-to websites, our stance Twitter files may not represent the stances in Twitter as a whole.

The fact that belief covariation, as measured by the first principle component loadings and scores, were similar after debotting and stance inference applied to tweets is encouraging for public health monitoring of Twitter. Re-sampling the data so that the number of topics about which individuals express strong opinions is similar to the distribution seen for elicited opinions further improves the agreement. While the exact elicited opinion distribution is unknown without a survey, our results suggest that down-sampling Twitter accounts that focus on a very limited number of topics and preferentially sampling accounts that tweet about a broader range of topics may lead to conclusions more representative of the population as a whole. Our PCA findings suggest that the overall pattern of how beliefs covary with one another are similar in nationally representative surveys and on Twitter if the Twitter data are suitably processed. We note that more work comparing belief covariation between survey and Twitter data is needed to understand how generalizable our findings are. Many of the topics included in our survey and Twitter data are highly polarized, and it is possible that belief covariation on highly polarized topics is more robust than less-polarized beliefs. Nevertheless, the results are promising. Twitter data could be a valid proxy measure for changes in the co-occurrence of beliefs among the population in response to interventions such as messaging campaigns by public health agencies.

## Supporting information

S1 Fig Flow diagram of processes used to create data file pairs. (EPS)

S2 Fig Scree plots for the topic data file pair. (TIF)

S3 Fig Scree plots for the stance data file pair. (TIF)

S4 Fig Scree plots for the limited topic data file pair. (TIF)

S5 Fig Scree plots for the resampled stance data file pair. (TIF)

S1 Table Belief items. Belief items from survey and corresponding twitter queries. In cases where the query is “n/a”, we were unable to develop a Twitter query with a low false positive rate that also returned a non-zero number of tweets.
(DOCX)

S2 Table Twitter query false positive rate. Two Coders (SAN and LJM) coded 20 tweets from each of three categories (Vaccine-related, political/conspiracy-related, and deep state). Tweets tagged with the 5 most common vaccine-related tags and 4 most common (non-deep state) political/conspiracy-related tags were coded. Deep State tweets were coded separately because they were far more common than other belief tags. Tweets were coded as agreeing with the belief, not agreeing with the belief, neutral, or not relevant (false positive). S2 Table shows the average of the false positive rate estimated by the two coders. The estimated false positive rate was zero for 6 out of 10 tags that were examined and 5 percent or lower for the remaining 4 belief tags.
(DOCX)

S3 Table Inter-rater reliability, coded tweets. This table shows the inter-rater reliability between the two coders (LJM and SAN).
(DOCX)

S4 Table Accuracy of stance prediction. Two coders (SAN and LJM) each coded the stance of tweets and URLs. The table shows the average agreement between stance coded in tweets and inferred using URL information for each of the two coders. While we initially coded 20 or more tweets for each tag, tweets without any url, with a url from a domain we did not code, and tweets with a url whose domain we coded as “neutral” were excluded from the analysis. These exclusions decreased our sample sizes. We do not show tag-level results for cases where we had fewer than 10 tweets for which we inferred stance. In the table a dash, “-”indicates that there were fewer than 10 tweets coded by each coder for which we were able to infer stance from a url. An asterisk following a reported number indicates that we are reporting the results for one coder only because we were able to infer stance on fewer than 10 of the other coders’ coded tweets.
(DOCX)

S5 Table Sensitivity analysis PC1 correlations. Here, we present the results of three sensitivity analysis. Results can be compared to those from Fig 2 in the main text. Alternate survey coding: In the main results, we code only the “Strongly agree is true” (6 on a Likert scale) and “Strongly disagree is true” (0 on a Likert scale) survey responses as non-neutral. In the alternate survey coding, we code all agree statements (4, 5, or 6 on the Likert scale) and all disagree statements (0, 1, or 2 on the Likert scale) as non-neutral. Strict bot removal: In the strict bot removal sensitivity analysis, we kept accounts with a CAP <0.2 instead of CAP<0.5, which corresponds to keeping accounts with less than a 20% probability of being a bot. Resample Twitter 100 times: In this sensitivity analysis, we re-sampled the Twitter data 100 times for the resampled stance data file pair, and report the inter-quartile range (IQR) of the results. We also find that the PC1 loadings are qualitatively similar to those reported in the main text. That is, the items on PC1 all loaded in the same direction for survey topic and survey limited topic data. Deep state loaded in a different direction from all other items in Twitter topic and limited topic analyses. In the stance and resampled stance Twitter and survey analyses, all items loaded in the same direction except for “Vaccines Benefit Public”.
(DOCX)

S6 Table Sensitivity analysis R squared. Here, we present the R squared results for the sensitivity analyses. Results can be compared to those from Fig 3 in the main text. Alternate survey coding: In the main results, we code only the “Strongly agree is true” (6 on a Likert scale) and “Strongly disagree is true” (0 on a Likert scale) survey responses as non-neutral. In the alternate survey coding, we code all agree statements (4, 5, or 6 on the Likert scale) and all disagree statements (0, 1, or 2 on the Likert scale) as non-neutral. Strict bot removal: In the strict bot removal sensitivity analysis, we kept accounts with a CAP <0.2 instead of CAP<0.5, which corresponds to keeping accounts with less than a 20% probability of being a bot. Resample Twitter 100 times: In this sensitivity analysis, we re-sampled the Twitter data 100 times for the resampled stance data file pair, and report the inter-quartile range (IQR) of the results.
(DOCX)

S1 File Twitter about 2020-04-03. (CSV)

S2 File Twitter stance 2020-04-03. (CSV)
