# Supplementary figures and images for: Comparing covariation among vaccine hesitancy and broader beliefs within Twitter and survey data
Source: PLoS One. 2020 Oct 8;15(10):e0239826. doi: 10.1371/journal.pone.0239826 (PMC7544030; doi:10.1371/journal.pone.0239826)

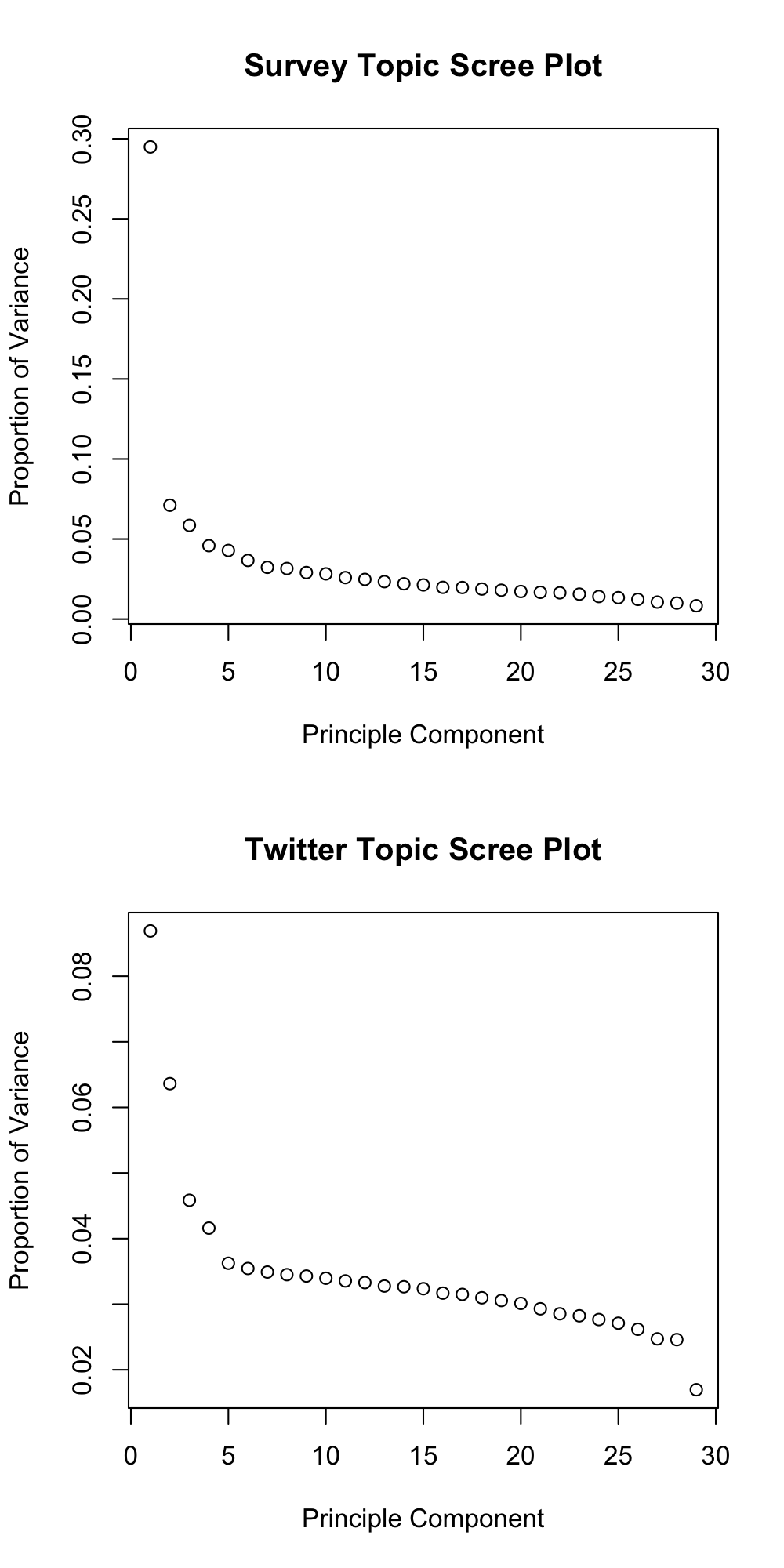

Supplement: S2 Fig — (TIF) [file pone.0239826.s002.tif]

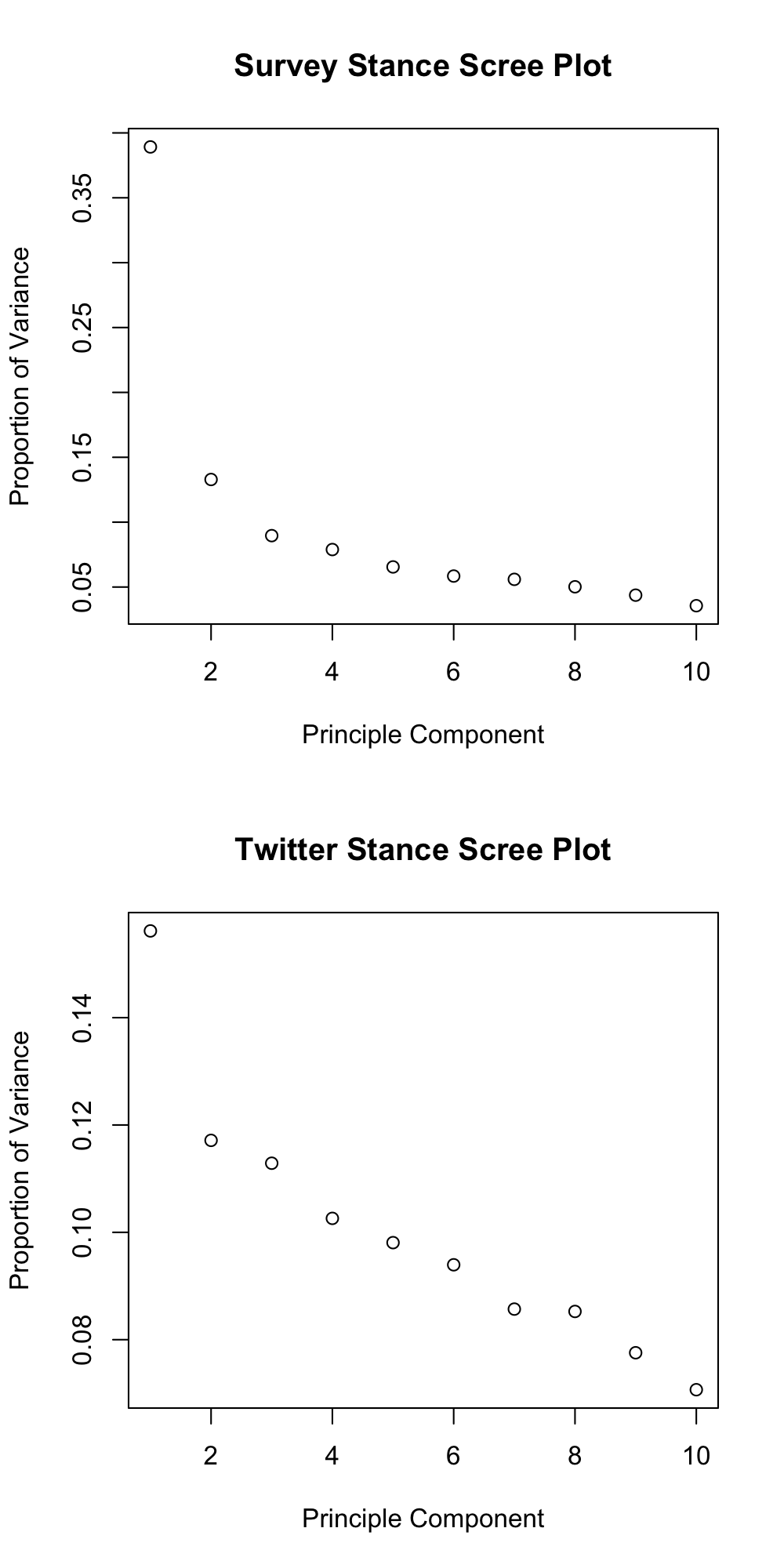

Supplement: S3 Fig — (TIF) [file pone.0239826.s003.tif]

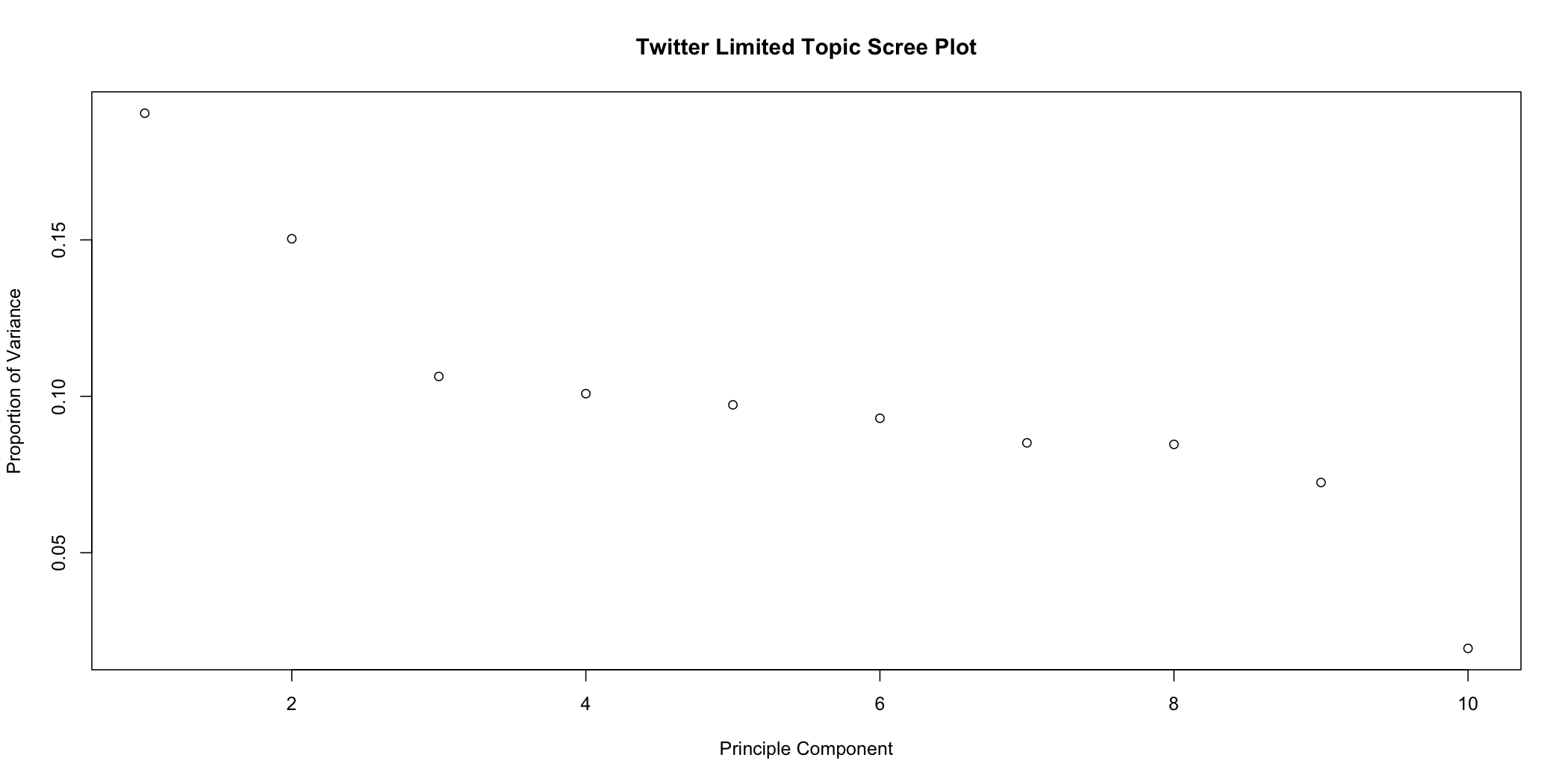

Supplement: S4 Fig — (TIF) [file pone.0239826.s004.tif]

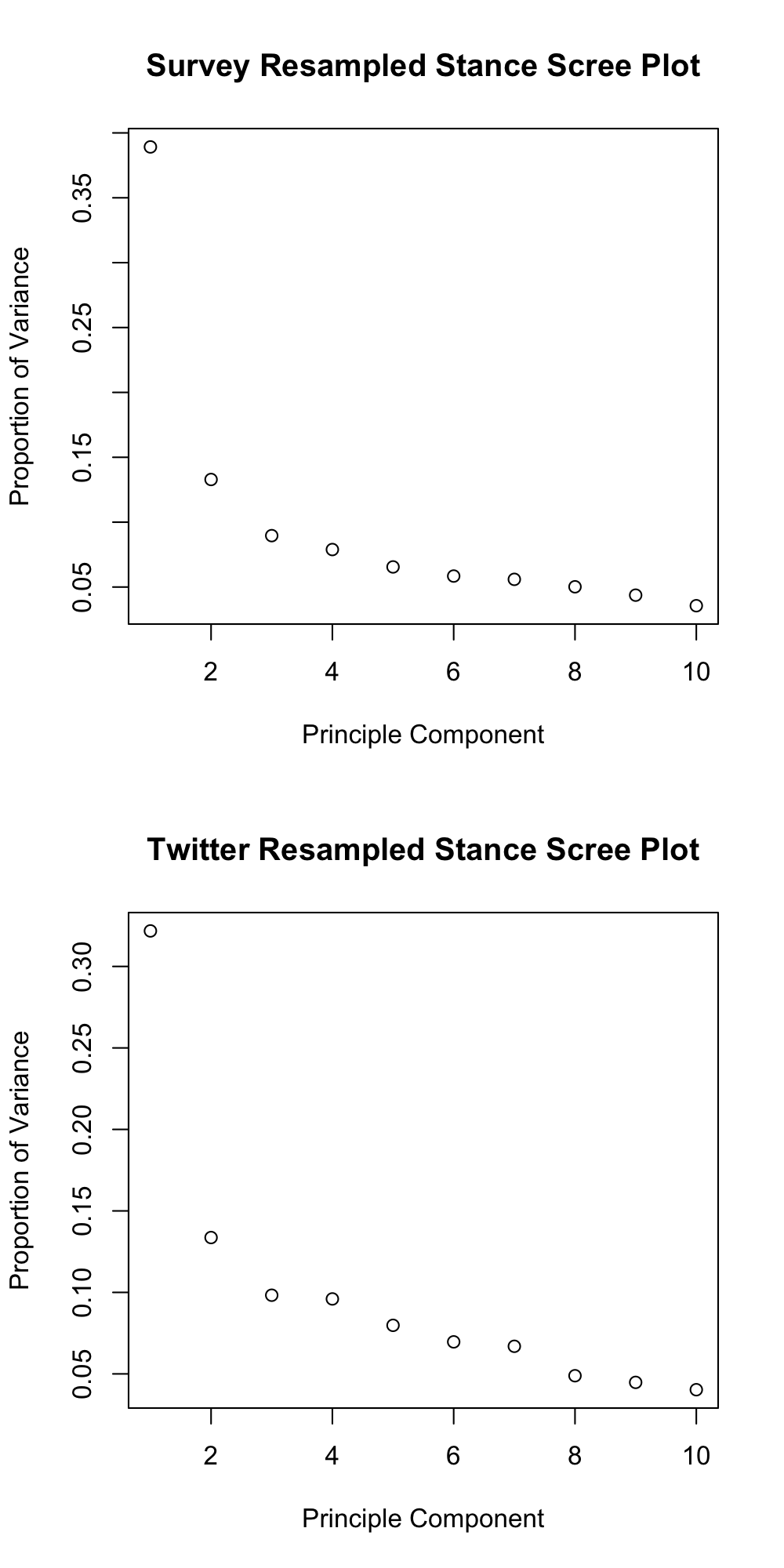

Supplement: S5 Fig — (TIF) [file pone.0239826.s005.tif]
