# Supplementary material for: Comparing covariation among vaccine hesitancy and broader beliefs within Twitter and survey data
Source: PLoS One. 2020 Oct 8;15(10):e0239826. doi: 10.1371/journal.pone.0239826 (PMC7544030; doi:10.1371/journal.pone.0239826)
Supplement: S1 Table — Belief items from survey and corresponding twitter queries. In cases where the query is “n/a”, we were unable to develop a Twitter query with a low false positive rate that also returned a non-zero number of tweets. (DOCX) [file pone.0239826.s006.docx]

| **In Topic data** | **Stance inferred for Twitter** | **Variable Name** | **Survey item** | **Twitter Query** |
| --- | --- | --- | --- | --- |
| Yes | Yes | JFK Assassination | The assassination of John F. Kennedy was part of a larger conspiracy | jfk AND conspiracy OR \"multiple shooters\" OR \"inside job\" OR ((killed OR assassinated) (cia OR fbi))) |
| Yes | No | Moon Landing Faked | At least some moon landings didn't actually happen but were faked productions in Hollywood | moon landing faked |
| Yes | No | Masons Secret Control | Most US leaders are controlled by societies like the Free Masons or Skull & Bones | (\"free mason\" OR \"free masons\" OR contains:freemason OR \"skull bones\"~2 OR \"secret society\" OR \"secret societies\") (\"united states\" OR us OR usa) contains:control |
| Yes | Yes | 9/11 Inside Job | The US government causes or let the 9/11 attack happen on purpose | (\"9/11\" OR 911) (truth OR truther OR \"inside job\" |
| Yes | No | 2016 Votes Manipulated | Votes counts in the 2016 Trump-Clinton election were manipulated | russia (change OR changed OR changing OR contains:manipulat) votes |
| Yes | Yes | Deep State | Elected US leaders are controlled by an unelected deep state within the US government | deep state |
| Yes | Yes | Birtherism | Barack Obama was not born in the United States | obama ((birth certificate (forged OR fake)) OR (born kenya)) |
| Yes | No | Immigration Plot | Immigration is part of a plot to decrease the number of white people | white genocide contains:immigra |
| No | No | CIA Created HIV | The CIA created HIV to kill African Americans | n/a |
| No | No | Vaccine Spread HIV Africa | HIV was spread by vaccination programs in Africa in the 1950s and 1960s | n/a |
| No | No | CIA Bin Laden Vaccination | The CIA conducted a fake vaccination program that collected DNA to find relatives of Osama bin Laden | n/a |
| Yes | Yes | Chemtrails | Chemtrails behind planes are being used by governments to poison the population | "chemtrails OR \"chem trails\"~2" |
| Yes | No | Pharma Tests Africa | Pharmaceutical companies are currently conducting medical experiments on Africans | contains:experiment (africa OR africans) (pharma OR \"pharmaceutical company\" OR \"pharmaceutical companies\" |
| Yes | No | Tuskegee Syphilis | During the Tuskegee syphilis event, government-funded scientists decided to not treat African Americans for syphilis | tuskegee syphilis |
| No | No | CDC Hides Side Effects | The CDC or other parts of the government are hiding evidence about vaccine side effects | n/a |
| Yes | No | Vaccines Created to Sterilize* | Governments have used vaccination campaigns to sterilize or harm populations they don't like | (contains:vaccin OR contains:vax) contains:steriliz |
| No | No | Vaccine Profit Motive | Vaccines don't prevent disease but instead are made primarily to sell more vaccines | n/a |
| Yes | Yes | Drs Hide Side Effects | Doctors and the health system aren't telling us everything they know about bad vaccine side effects | (contains:vaccin OR contains:vax) (\"side effect\" OR \"side effects\" OR injury) |
| Yes | Yes | Vaccines Benefit Public | Generally speaking, vaccines are a benefit for public health | (contains:vaccin OR Vax) contains:benefit contains:outweigh OR vaccinessavelives |
| Yes | No | Drs Serve Best Interest | Doctors have their patients' best interest at heart when they recommend vaccines | doctors best interest (contains:vaccin OR contains:vax) |
| Duplicate | No | HPV Vacc Causes Sterility | The HPV (Human Papilloma Virus) vaccine can cause sterility | (contains:vaccin OR contains:vax) contains:steriliz |
| Yes | No | HPV Vacc Causes Promiscuity | The HPV vaccine can make teenagers more promiscuous | ( contains:vaccin or vax) AND (contains:promiscu OR sex) |
| Yes | No | Flu Vaccine Causes Flu | The flu vaccine can give you the flu | \"flu from\" (contains:vaccin OR contains:vax)" |
| Yes | Yes | MMR Autism | The MMR (Measles, Mumps, Rubella) vaccine can cause Autism | contains:vaccin contains:autis -(contains:\"@vaccin\" OR contains:\"@autis\")" |
| Yes | Yes | Vaccines Cause SIDS | Childhood vaccines can cause SIDS (Sudden Infant Death Syndrome) | contains:vaccine or contains:vax (sids OR \"sudden infant death syndrome\") |
| Yes | No | Vaccine Overload | Multiple vaccines at one time can overload a child's immune system | contains:vaccin overload \"immune system\" |
| Yes | No | Guillian-Barre | Vaccines can cause Guillain-Barre syndrome | lang:en (contains:vaccin OR contains:vax) guillian-barre" |
| Yes | Yes | Vaccines Cause Asthma | Vaccines can cause asthma or allergies | contains:vaccin (asthma OR contains:allerg) -(\"allergic reaction\"~2)" |
| Yes | No | Vaccines Cause ADHD | Vaccines can cause hyperactivity or other behavioral problems | contains:vaccine (contains:hyper OR adhd OR \"behavioral problem\" OR \"behavioral problems\")" |
| Yes | No | Vaccines Kill Viruses | Vaccines work by killing viruses in your body | contains:vaccin contains:kill contains:virus |
| Yes | No | Vaccines Build Immunity | Vaccines cause your body to build an immunity against a disease | contains:vaccin (build OR builds OR strengthen OR strengthens) contains:immun |
| Yes | No | Vaccines Net Benefit | The benefits of vaccines outweigh the risks | contains:vaccin contains:benefit contains:outweigh |
| Yes | No | Natural Immunity | Natural immunity from getting diseases is better than the immunity you get from vaccines | contains:vaccin natural immunity OR (normal (disease OR measles OR flu)) |
| Yes | No | Vaccines Insignificant | Vaccines have not been significant contributors to most 20th century declines of vaccine-preventable disease; these declines were caused primarily by other public health measures | contains:vaccin sanitation |
| Yes | No | Vaccines Stop Working | Vaccines stop working well if you keep getting them | contains: vaccine (stop OR stops) working |
