# Supplementary material for: Comparing covariation among vaccine hesitancy and broader beliefs within Twitter and survey data
Source: PLoS One. 2020 Oct 8;15(10):e0239826. doi: 10.1371/journal.pone.0239826 (PMC7544030; doi:10.1371/journal.pone.0239826)
Supplement: S2 Table — Two Coders (SAN and LJM) coded 20 tweets from each of three categories (Vaccine-related, political/conspiracy-related, and deep state). Tweets tagged with the 5 most common vaccine-related tags and 4 most common (non-deep state) political/conspiracy-related tags were coded. Deep State tweets were coded separately because they were far more common than other belief tags. Tweets were coded as agreeing with the belief, not agreeing with the belief, neutral, or not relevant (false positive). S2 Table shows the average of the false positive rate estimated by the two coders. The estimated false positive rate was zero for 6 out of 10 tags that were examined and 5 percent or lower for the remaining 4 belief tags. (DOCX) [file pone.0239826.s007.docx]

| Belief Tag | Estimated False Positive Rate (Percent) |
| --- | --- |
| Vaccines Benefit Public | 1 |
| MMR Autism | 0 |
| Drs Hides Side Effects | 0 |
| Vaccines Cause Asthma | 0 |
| Vaccines Cause SIDS | 0 |
| Chemtrails | 5 |
| Birtherism | 0 |
| 9/11 Inside Job | 2.5 |
| JFK Assassination | 2.5 |
| Deep State | 0 |
