# Supplementary material for: Comparing covariation among vaccine hesitancy and broader beliefs within Twitter and survey data
Source: PLoS One. 2020 Oct 8;15(10):e0239826. doi: 10.1371/journal.pone.0239826 (PMC7544030; doi:10.1371/journal.pone.0239826)
Supplement: S3 Table — This table shows the inter-rater reliability between the two coders (LJM and SAN). (DOCX) [file pone.0239826.s008.docx]

|  | Percent Agreement | Cohen’s κ |
| --- | --- | --- |
| Vaccines Benefit Public | 95 | 0.84 |
| MMR Autism | 71 | 0.57 |
| Drs Hide Side Effects | 100 | NaN |
| Vaccines Cause Asthma | 100 | NaN |
| Vaccines Cause SIDS | 96 | 0 |
| Chemtrails | 83 | 0.71 |
| Birtherism | 78 | 0.62 |
| 9/11 Inside Job | 83 | 0.74 |
| JFK Assassination | 75 | 0.48 |
| Deep State | 82 | 0.45 |
