# Supplementary material for: Comparing covariation among vaccine hesitancy and broader beliefs within Twitter and survey data
Source: PLoS One. 2020 Oct 8;15(10):e0239826. doi: 10.1371/journal.pone.0239826 (PMC7544030; doi:10.1371/journal.pone.0239826)
Supplement: S4 Table — Two coders (SAN and LJM) each coded the stance of tweets and URLs. The table shows the average agreement between stance coded in tweets and inferred using URL information for each of the two coders. While we initially coded 20 or more tweets for each tag, tweets without any url, with a url from a domain we did not code, and tweets with a url whose domain we coded as “neutral” were excluded from the analysis. These exclusions decreased our sample sizes. We do not show tag-level results for cases where we had fewer than 10 tweets for which we inferred stance. In the table a dash, “-”indicates that there were fewer than 10 tweets coded by each coder for which we were able to infer stance from a url. An asterisk following a reported number indicates that we are reporting the results for one coder only because we were able to infer stance on fewer than 10 of the other coders’ coded tweets. (DOCX) [file pone.0239826.s009.docx]

|  | Average % agreement | Average Cohen’s  |
| --- | --- | --- |
| Vaccines Benefit Public | 98 | 0.95 |
| MMR Autism | 58.5 | 0.38 |
| Drs Hide Side Effects | - | - |
| Vaccines Cause Asthma | - | - |
| Vaccines Cause SIDS | 92* | 0.85* |
| Chemtrails | - | - |
| Birtherism | - | - |
| 9/11 Inside Job | 66* | 0.42* |
| JFK Assassination | - | - |
| Deep State | 78 | 0.275 |
