# Supplementary material for: Comparing covariation among vaccine hesitancy and broader beliefs within Twitter and survey data
Source: PLoS One. 2020 Oct 8;15(10):e0239826. doi: 10.1371/journal.pone.0239826 (PMC7544030; doi:10.1371/journal.pone.0239826)
Supplement: S5 Table — Here, we present the results of three sensitivity analysis. Results can be compared to those from Fig 2 in the main text. Alternate survey coding: In the main results, we code only the “Strongly agree is true” (6 on a Likert scale) and “Strongly disagree is true” (0 on a Likert scale) survey responses as non-neutral. In the alternate survey coding, we code all agree statements (4, 5, or 6 on the Likert scale) and all disagree statements (0, 1, or 2 on the Likert scale) as non-neutral. Strict bot removal: In the strict bot removal sensitivity analysis, we kept accounts with a CAP <0.2 instead of CAP<0.5, which corresponds to keeping accounts with less than a 20% probability of being a bot. Resample Twitter 100 times: In this sensitivity analysis, we re-sampled the Twitter data 100 times for the resampled stance data file pair, and report the inter-quartile range (IQR) of the results. We also find that the PC1 loadings are qualitatively similar to those reported in the main text. That is, the items on PC1 all loaded in the same direction for survey topic and survey limited topic data. Deep state loaded in a different direction from all other items in Twitter topic and limited topic analyses. In the stance and resampled stance Twitter and survey analyses, all items loaded in the same direction except for “Vaccines Benefit Public”. (DOCX) [file pone.0239826.s010.docx]

|  | Topic | Stance | Limited Topic | Resampled Stance |
| --- | --- | --- | --- | --- |
| Alternate survey coding | 0.34 | 0.91 | 0.12 | 0.99 |
| Strict bot removal | 0.56 | 0.90 | 0.29 | 0.94 |
| Resample Twitter 100 times | n/a | | | IQR: [0.93,0.95] |
