# Supplementary material for: Comparing covariation among vaccine hesitancy and broader beliefs within Twitter and survey data
Source: PLoS One. 2020 Oct 8;15(10):e0239826. doi: 10.1371/journal.pone.0239826 (PMC7544030; doi:10.1371/journal.pone.0239826)
Supplement: S6 Table — Here, we present the R squared results for the sensitivity analyses. Results can be compared to those from Fig 3 in the main text. Alternate survey coding: In the main results, we code only the “Strongly agree is true” (6 on a Likert scale) and “Strongly disagree is true” (0 on a Likert scale) survey responses as non-neutral. In the alternate survey coding, we code all agree statements (4, 5, or 6 on the Likert scale) and all disagree statements (0, 1, or 2 on the Likert scale) as non-neutral. Strict bot removal: In the strict bot removal sensitivity analysis, we kept accounts with a CAP <0.2 instead of CAP<0.5, which corresponds to keeping accounts with less than a 20% probability of being a bot. Resample Twitter 100 times: In this sensitivity analysis, we re-sampled the Twitter data 100 times for the resampled stance data file pair, and report the inter-quartile range (IQR) of the results. (DOCX) [file pone.0239826.s011.docx]

|  | Topic | | Stance | | Limited Topic | | Resampled Stance | |
| --- | --- | --- | --- | --- | --- | --- | --- | --- |
|  | Survey | Twitter | Survey | Twitter | Survey | Twitter | Survey | Twitter |
| Alternate survey coding | 0.86 | 0.45 | 0.96 | 0.70 | 0.84 | 0.26 | 0.99 | 0.99 |
| Strict bot removal | 0.92 | 0.52 | 0.95 | 0.68 | 0.98 | 0.27 | 0.97 | 0.98 |
| Resample Twitter 100 times | n/a | | | | | | IQR: [0.97, 0.98] | IQR: [0.98, 0.99] |
